# Effectiveness of Adjuvant Chemo- and Radiotherapy in Thymic Carcinoma Stage II: A Systematic Review and Meta-Analysis

**DOI:** 10.1177/10732748241292781

**Published:** 2024-10-17

**Authors:** Ahmad Nazzal, Josef Yayan, Christian Biancosino, Seyed Vahid Tabatabaei, Khosro Hekmat

**Affiliations:** 1 Department of Internal Medicine, Division of Pulmonary, Allergy and Sleep Medicine, HELIOS Clinic Wuppertal, 489031 Witten/Herdecke University, Witten, Germany; 2 Department of Thoracic Surgery, HELIOS Clinic Wuppertal, 489031 Witten/Herdecke University, Witten, Germany; 3 Department of Internal Medicine, Division of Pulmonary, 39945 Rein-Maas Klinikum, Academic Teaching Hospital of the RWTH Aachen University, Würselen, Germany; 4 Department of Cardiothoracic Surgery, University Clinic of Cologne, Cologne, Germany

**Keywords:** thymoma, thymic carcinoma, chemotherapy, radiotherapy, survival rate, adjuvant therapy

## Abstract

**Background:**

Thymic carcinoma is a rare tumor arising from the epithelial thymic tissue, yet among mediastinal tumors, it is the most common malignant entity. Thymic carcinoma often causes no symptoms and is incidentally discovered. Adjuvant radiotherapy is recommended, particularly in cases of incomplete resection and for stages III and IV, based on current guidelines and existing literature. In stage II (Masaoka-Koga system), the role of chemotherapy remains controversial, particularly in cases of incomplete resection. Therefore, this study aims to assess the effectiveness of adjuvant chemotherapy compared to adjuvant radiotherapy in the treatment of stage II (Masaoka-Koga system) thymic carcinoma after surgery.

**Methods:**

A comprehensive literature search was conducted in the Embase, Cochrane Central Register of Controlled Trials (CENTRAL), and MEDLINE/PubMed databases for relevant studies published through April 30^th^, 2023.

**Results:**

The review identified a total of 101 studies in the Embase, Cochrane Library, and MEDLINE/PubMed databases. Of these, only eight retrospective studies met the inclusion criteria and were included in the meta-analysis. These eight studies encompassed a total of 323 patients with stage II (Masaoka-Koga system) thymic carcinoma, with an average patient age of 53.8 ± 5.0 years. There was no significant difference in the number of R0 resections between patients receiving adjuvant chemotherapy and those receiving adjuvant radiotherapy (*P* = 0.82). Patients who received adjuvant radiotherapy had a higher rate of recurrence, but this difference was not statistically significant (*P* = 0.93). The meta-analysis synthesized five-year overall survival data, with a combined hazard ratio (HR) of 0.881 (95% CI: 0.463 to 1.299), indicating no statistically significant difference between the treatment groups.

**Conclusions:**

Although the results were not statistically significant, the findings suggest that adjuvant chemotherapy might be associated with a more favorable outcome for patients with stage II (Masaoka-Koga system) thymic carcinoma compared to adjuvant radiotherapy.

## Introduction

The thymus gland is located in the mediastinum and is involved in the development of the immune system up to puberty.^1^ The thymus typically undergoes physiological involution after puberty, meaning it shrinks and is largely replaced by fatty tissue.^2^ Tumors can develop in the thymus in any age group. Thymic epithelial tumors most commonly appear between the ages of 50 and 60.^3^ Thymic epithelial tumors often cause no symptoms and are incidentally discovered.^4^ Symptoms usually arise from infiltration of surrounding structures.^5^ These symptoms include breathing difficulties, coughing, hoarseness, chest pain, and heart failure.^6^ Paraneoplastic syndromes, especially myasthenia gravis, are particularly characteristic.^7^ While many thymic epithelial tumors are benign, thymomas can also be malignant.^8^ Thymic carcinoma is a rare and highly malignant tumor that originates in the thymus gland, which is essential for the development and maturation of T-cells.^9^ The histology grade of thymic epithelial tumors is a critical factor that significantly influences both prognosis and treatment strategies. Thymic carcinoma is estimated to account for less than 1% of all malignancies and has a prevalence of 0.15 per 100 000 people per year worldwide.^10^ The disease is challenging to diagnose, with nonspecific symptoms, and has a poor prognosis, with a five-year survival rate ranging from 25% to 67%, depending on the stage at diagnosis.^11^ Malignant locally aggressively growing thymic carcinomas can spread through the lymphatic system.^12^ Surgery, chemotherapy, radiotherapy, and hormone therapy are used to treat malignant thymic epithelial tumors.^13^ Hormone therapy is not a standard treatment for thymic epithelial tumors. Hormone therapy, such as with somatostatin, is used in specific cases, not routinely. The type of therapy depends on various factors, such as the stage of the thymic carcinoma, the type of cell to which the thymic carcinoma has been assigned, the general condition of the patient, the complete surgical resection of the thymic carcinoma, and whether it is a recurrence.^4^ The standard therapy involves surgical removal of the thymic carcinoma.^14^ During surgery, the entire thymus, the lymph nodes and the fat and connective tissue of the anterior mediastinum are removed.^15^ In stage I, according to Masaoka, after complete removal of the tumor, no follow-up treatment is required.^16^ In all other stages, adjuvant radiotherapy is used to support the operation^17^ and can significantly reduce the likelihood of recurrence after surgery.^17^ Combined radiochemotherapy is also possible^18^ and is also used when an operation cannot be performed, eg, because the patient is too weak, has other serious illnesses or the tumor is extensive.^18^ In many cases, hormone therapy with somatostatin can be given prior to the operation.^19^ Hormone therapy allows a reduction in the tumor mass in advanced tumors, simplifying the subsequent operation.^19^ In stage II (Masaoka-Koga system), adjuvant therapy is controversial and should be performed at least in the case of incomplete resection.^20^ In the case of locally advanced thymic carcinoma that cannot be completely surgically and removed, chemotherapy before an operation can reduce the size of the tumor and enable its subsequent complete surgical removal.^21^ If there is a limited metastasis on one half of the chest, a combination treatment of surgery and chemotherapy can be useful in individual cases.^22^ Chemotherapy is required when metastases are also found outside the chest.^23^ In principle, thymic carcinomas are sensitive to chemotherapy.^3^ However, convincing data that would support the routine use of chemotherapy is currently unavailable.^23^ To address these uncertainties, the present work clarifies the benefits of adjuvant chemotherapy and radiotherapy in stage II (Masaoka-Koga system) thymic carcinoma. The present work also includes a literature search to identify the relevant studies and collect the relevant data on patients with stage II (Masaoka-Koga system) thymic carcinoma who were evaluated up to April 30^th^, 2023, and treated with adjuvant chemotherapy and radiotherapy. The primary objective of this study is to analyze the impact of adjuvant chemotherapy and adjuvant radiotherapy on the overall survival and disease-free survival of patients with stage II (Masaoka-Koga system) thymic carcinoma. Secondary objectives include evaluating prognostic factors associated with survival outcomes. The results of this study provide valuable insights into the effectiveness of adjuvant therapy in stage II (Masaoka-Koga system) thymic carcinoma and may guide clinical decision-making in the management of this rare and aggressive malignancy. Furthermore, this study identifies gaps in knowledge and informs future research directions in the treatment of thymic carcinoma.

## Materials and Methods

### Study Selection and Eligibility Criteria

Adhering to MOOSE guidelines and the PRISMA statement, our review focused on stage II (Masaoka-Koga system) thymic carcinoma patients treated with adjuvant chemotherapy and radiotherapy (Supplemental Material).^24^ Inclusion criteria were: (1) studies published until April 30^th^, 2023; (2) inclusive of patient age and treatment outcome data; (3) conducted on human subjects; (4) written in English and published in peer-reviewed journals. Exclusion criteria encompassed: (1) studies not specifically addressing stage II (Masaoka-Koga system) thymic carcinoma; (2) absence of detailed treatment outcome data; (3) non-research articles like reviews, editorials, or case reports.^25^ This systematic review and meta-analysis has been registered with the International Prospective Register of Systematic Reviews (PROSPERO) to ensure transparency and methodological rigor. The review protocol was registered prior to the data extraction and analysis phase to avoid any potential biases and to maintain a structured research process. The registration number for this review is CRD42024590733. Registration with PROSPERO helps to minimize duplication of efforts, provides a public record of the review methodology, and promotes the dissemination of high-quality evidence in the field of thymic carcinoma treatment.

### Search Strategy and Data Sources

A systematic search was conducted in Embase, CENTRAL, and MEDLINE/PubMed using specific keywords related to thymic carcinoma, such as 'Thymic squamous cell carcinoma,' 'Masaoka-Koga stage,' 'Surgery types,' 'Chemotherapy,' 'Radiotherapy,' and 'Prognosis'.^26^ To ensure comprehensive coverage, we manually reviewed references in identified articles and relevant review papers. The search process was documented for reproducibility and transparency.

### Data Extraction and Quality Assessment

Data extraction focused on patient demographics, carcinoma stage, types of surgical procedures, specific details of chemotherapy regimens (type, duration) and radiotherapy treatments (type, doses, and volumes), and outcomes such as recurrence and survival rates.^27^ For chemotherapy, we recorded the type of drugs used, the dosage (in mg/m^2^), the number of cycles, and the treatment schedule. For radiotherapy, we detailed the type of radiotherapy, the total dose (in Gy), the fractionation schedule, and the treated volume.

Quality assessment was conducted using the ROBINS-I tool, evaluating aspects such as confounding, selection bias, and intervention classification.^28^ Any discrepancies in data extraction or quality assessment were resolved through discussion and consensus between two independent reviewers.

### Synthesis Without Meta-Analysis vs Meta-Analysis

Given the significant heterogeneity among the studies, we applied the Synthesis Without Meta-analysis (SWiM) criteria to provide a narrative synthesis, complementing the traditional meta-analysis. While the meta-analysis offers a quantitative summary of the effects, SWiM allows for deeper insight into the qualitative and contextual differences between studies, particularly when dealing with diverse or complex datasets.^29^

### Statistical Analysis using Python

Statistical analyses were performed using Python, known for its robust libraries in data processing and statistical analysis. We calculated the standard error and variance of the log-transformed Hazard Ratios using Python’s statistical functions. Weighted average effect sizes were computed with inverse variance weighting. Python’s advanced statistical libraries facilitated the calculation of heterogeneity metrics, including the Q-Statistic and I^2^ Statistic. Data visualization tools in Python were utilized to create a forest plot for comparing survival outcomes and a funnel plot for assessing publication bias, aligning with the methodologies suggested by Sterne and Egger.

### Definitions and Calculations

#### Overall Survival (OS)

The time from the date of surgery to the date of death from any cause.

#### Recurrence-Free Survival (RFS)

The time from the date of surgery to the date of the first documented recurrence of thymic carcinoma or death from any cause, whichever occurred first.

#### Percentage of Patients with Recurrence at 5 Years

The proportion of patients who experienced a recurrence of thymic carcinoma within 5 years after surgery.

#### Median Recurrence-Free Survival (RFS)

The median time from the date of surgery to the date of the first documented recurrence of thymic carcinoma or death from any cause, whichever occurred first.

#### 5-Year Overall Survival (OS) Rate

The percentage of patients who were still alive 5 years after surgery.

The recurrence-free survival (RFS) and overall survival (OS) times were calculated using the Kaplan-Meier method. Hazard ratios (HR) for survival outcomes were extracted from the studies or calculated when not provided.^30^ We used the log-rank test to compare survival distributions between different treatment groups. Statistical significance was set at *P* < 0.05.

## Results

**Figure 1.**
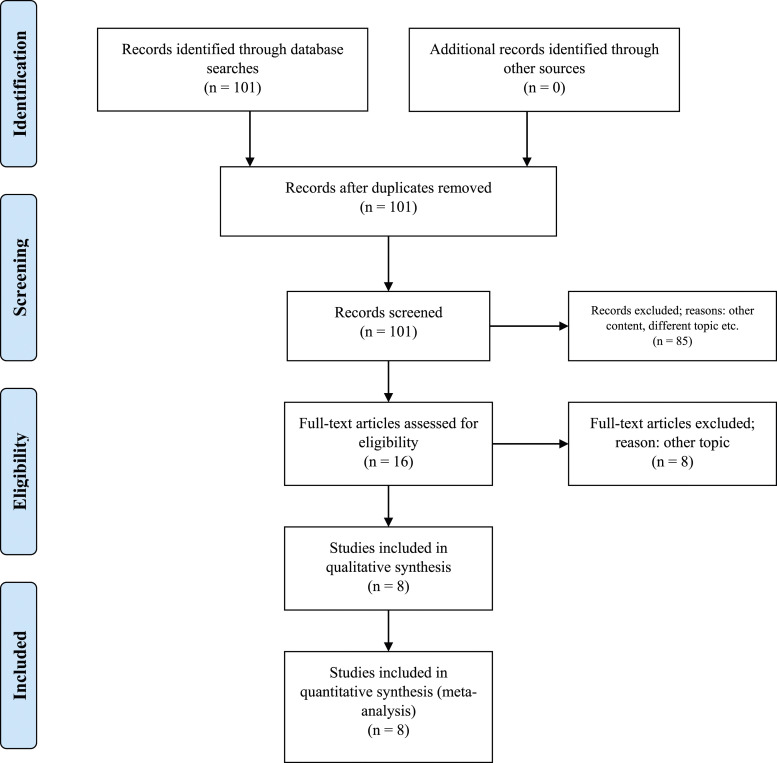
Preferred reporting items for systematic reviews and meta-analyses (PRISMA) 2009 flow chart of data collection after identifying suitable studies. Entering the search criteria into the Embase, CENTRAL, and MEDLINE/PubMed search engines yielded a total of 101 studies for the period ending April 30^th^, 2023. A critical review of these published studies identified eight studies that met the inclusion criteria for the present meta-analysis.

**Figure 2.**
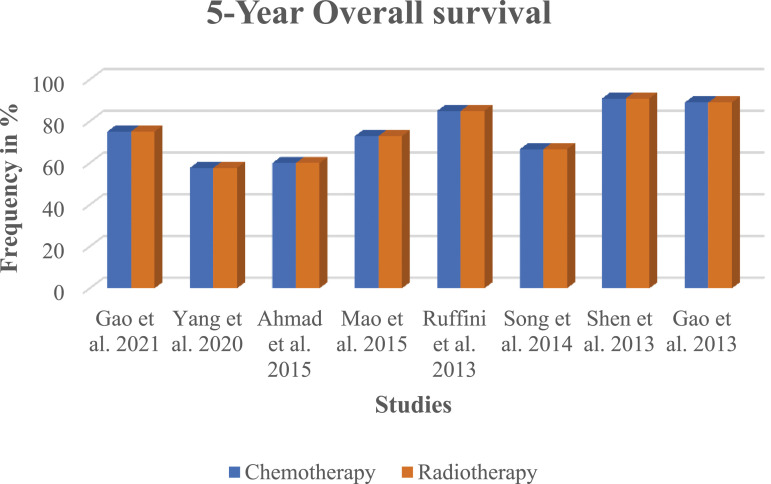
Comparison of the frequency of five-year overall survival of patients with stage II (Masaoka-Koga system) thymic carcinoma between adjuvant chemotherapy and adjuvant radiotherapy among the different studies.

**Figure 3.**
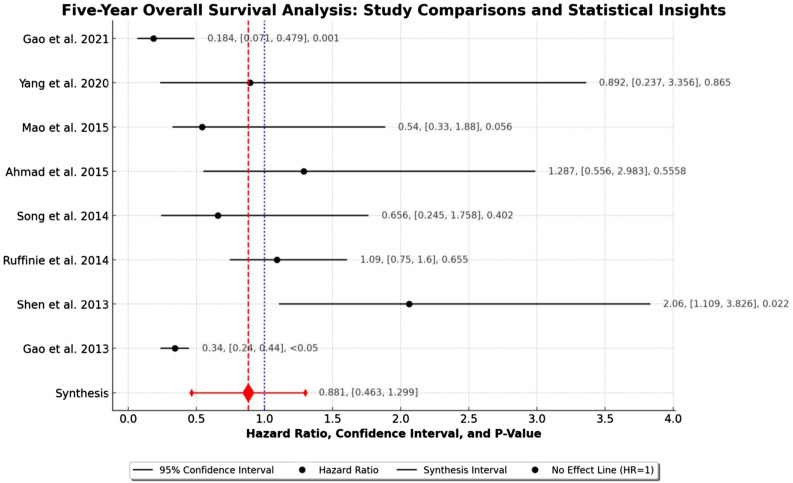
Overall, the forest plot shows a mixture of effects across the studies. Some studies suggest a protective effect (HR < 1), while others indicate increased risk (HR > 1). The significance of these effects varies, as reflected by the *P*-values. The vertical line at HR = 1 in the plot is a point of no effect, where values to its left indicate a protective effect and to its right indicate increased risk. The width of the CI in each study reflects the precision of the estimate.

**Figure 4.**
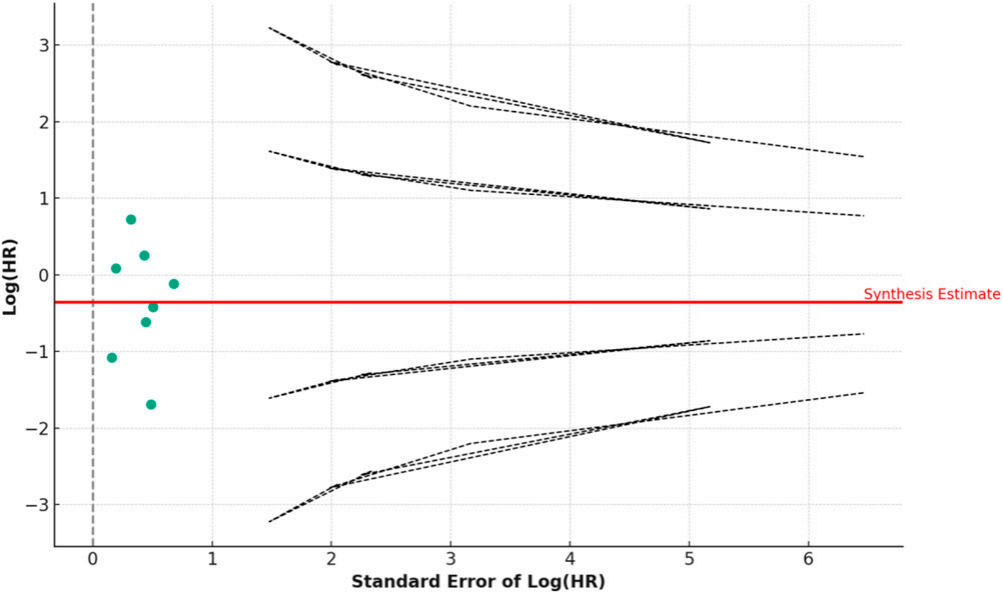
The funnel plot illustrates the distribution of studies in the meta-analysis. Symmetry around the average log (HR) is crucial in assessing potential biases and heterogeneity among the included studies.

Entering the search criteria in the Embase, Cochrane Library, and MEDLINE/PubMed databases for the period ending April 30^th^, 2023, yielded a total of 101 studies across the databases. Eight retrospective studies met the inclusion criteria for this meta-analysis (Table 1 and Figure 1). An examination of these eight studies revealed a total of 323 patients with stage II (Masaoka-Koga system) thymic carcinoma, of which 163 (50.5%, 95% CI 44.9%-56.1%) patients received adjuvant chemotherapy and 160 (49.5%, 95% CI 44.0%-55.1%) patients received adjuvant radiotherapy. The average age of all examined patients with stage II (Masaoka-Koga system) thymic carcinoma was 53.8 ± 5.0 years. The chemotherapy regimens included cisplatin, etoposide, and cyclophosphamide with doses ranging from moderate to high intensity, depending on the specific cancer type and patient tolerance, typically varying between 50-100 mg/m^2^ for cisplatin, 80-120 mg/m^2^ for etoposide, and 500-1000 mg/m^2^ for cyclophosphamide. The chemotherapy regimen involved administering cycles that varied in number from 4 to 6, with each cycle being administered every 3 weeks. Radiotherapy involved a total dose of 45-60 Gy, delivered in daily fractions of 1.8-2 Gy, targeting the tumor bed and regional lymph nodes. The number of R0 resections did not differ between patients with stage II (Masaoka-Koga system) thymic carcinoma receiving adjuvant chemotherapy and those receiving adjuvant radiotherapy (*P* = 0.82; Table 2). The patients with stage II (Masaoka-Koga system) thymic carcinoma and adjuvant radiotherapy had, on average, more recurrences, although this was not statistically significant (*P* = 0.93; Table 2). The recurrence-free survival (RFS) time was increased in patients with stage II (Masaoka-Koga system) thymic carcinoma with adjuvant chemotherapy compared to adjuvant radiotherapy, although this difference was not statistically significant. (*P* = 0.47; Table 2). Likewise, adjuvant chemotherapy increased the 5-year overall survival rate for patients with stage II (Masaoka-Koga system) thymic carcinoma compared to adjuvant radiotherapy (*P* = 0.99; Table 2 and Figure 2). Our analysis revealed that patients who received adjuvant chemotherapy had a longer recurrence-free survival (RFS) compared to those who received adjuvant radiotherapy, although this difference was not statistically significant (*P* = 0.47). Similarly, the five-year overall survival (OS) rate was higher in the chemotherapy group, but again, the difference did not reach statistical significance (*P* = 0.99). These findings suggest a potential, though not definitive, advantage of chemotherapy in extending survival outcomes, which warrants further investigation. The non-significant *P*-values indicate that there was no strong evidence to suggest a statistically significant difference between the treatment groups in this meta-analysis. The forest plot analysis illustrates a range of hazard ratios over a five-year period, reflecting diverse outcomes across studies. Gao et al (2021) reported the most protective effect with an HR of 0.184 (95% CI: [0.071, 0.479], *P* = 0.001). In contrast, Shen et al (2013) found a significantly increased risk with an HR of 2.06 (95% CI: [1.109, 3.826], *P* = 0.02). The synthesized data from all studies yielded an HR of 0.881 (95% CI: [0.463, 1.299]), suggesting no overall significant effect. The variability in individual study outcomes underscores the complex nature of survival analysis and the necessity of considering individual study contexts when interpreting the meta-analysis. The evaluation of the study quality assessment tool according to ROBIN-I yielded 4 (50%) studies with a low risk of bias: 2 (25%) studies with a moderate risk of bias, and 2 (25%) studies with a serious risk of bias (Table 3). The overall survival effect varies across studies. While some studies like Gao et al (both entries) and Shen et al show statistically significant effects (either improvement or detriment), others like Yang et al, Ahmad et al, Mao et al, Ruffinie et al, and Song et al do not show significant effects. This variation could be due to different study designs, populations, interventions, or other factors (Figure 3). The calculated average effect size across all studies is 0.795. The Q-statistic, which measures the total variance among the studies, is 30.34. Based on this, the I^2^ value, indicating the proportion of variation among studies that is not due to chance, is 76.93%. This suggests a high level of heterogeneity among the studies. The funnel plot illustrates the distribution of studies in the meta-analysis. Symmetry around the average log (HR) is crucial in assessing potential biases and heterogeneity among the included studies (Figure 4).

**Table 1.** All Studies Found for This Meta-Analysis Were Grouped by Study Number, Reference Number, Citation, Country of Main Author, Total Number of Patients, Number of Patients Considered for This Study, and Funding.

Study number	Author	Year of publication	Reference number	Citation	Country of main author	Total number of study participants	No. of participants included in the study	Funding
1	Gao et al.	2021	31	Adjuvant chemotherapy improves survival outcomes after complete resection of thymic squamous cell carcinoma: a retrospective study of 116 patients. Interact Cardiovasc Thorac Surg. 2021 Oct 4; 33(4): 550-556	China	116	44	No funding
2	Yang et al.	2020	32	Thymic Squamous Cell Carcinoma: A Population-Based Surveillance, Epidemiology, and End Result Analysis. Front Oncol. 2020 Dec 22; 10: 592023	China	276	55	Yes, Natural Science Foundation of China
3	Ahmad et al.	2015	33	Thymic carcinoma outcomes and prognosis: results of an international analysis. J Thorac Cardiovasc Surg. 2015 Jan; 149(1): 95-100, 101.e1-2	United States of America	1042	24	No funding
4	Mao et al.	2015	34	Treatment and survival analyses of completely resected thymic carcinoma patients. Onco Targets Ther. 2015 Sep 9; 8: 2503-7	China	54	41	No funding
5	Ruffini et al.	2014	35	European Association of Thoracic Surgeons (ESTS) Thymic Working Group. Tumours of the thymus: a cohort study of prognostic factors from the European Society of Thoracic Surgeons database. Eur J Cardiothorac Surg. 2014 Sep; 46(3): 361-8	Italy	2151	100	No funding
6	Song et al.	2014	36	Outcomes after surgical resection of thymic carcinoma: A study from a single tertiary referral centre. Eur J Surg Oncol. 2014 Nov; 40(11): 1523-7	China	76	29	No funding
7	Shen at al.	2013	37	Long-term survival in thymic epithelial tumors: a single-center experience from China. J Surg Oncol. 2013 Feb; (2): 167-72	China	115	14	No funding
8	Gao et al.	2013	38	Outcome of multimodality treatment for 188 cases of type B3 thymoma. J Thorac Oncol. 2013 Oct; 8(10): 1329-34	China	188	16	Yes, Research grant from Shanghai Jiaotong University and Science and Technology Commission of Shanghai, China and the National Natural Science Foundation of China

**Table 2.** The Presentation of the Studies Evaluated Here Compare Patients With Stage II (Masaoka-Koga System) Thymic Carcinoma According to the Number of Chemotherapy and Radiotherapy Treatments, Mean Age, Number of R0 Resections, Number of Recurrences, Number of Recurrence-free Times, and Overall Survival time. Total Number Chemotherapy/Radiotherapy: Number of Patients Receiving Chemo or Radiotherapy. Percentage of Patients With Recurrence at 5 Years (Chemo-/Radiotherapy): The Proportion of Patients Who Experienced a Recurrence of Thymic Carcinoma Within 5 years After Surgery. Recurrence-Free Survival: The Median Time From the Date of Surgery to the Date of the First Documented Recurrence of Thymic Carcinoma or Death From Any Cause, Whichever Occurred First. 5-Year Overall Survival Rate: The Percentage of Patients Who Were Still Alive 5 years After Surgery.

Studies	Total number Chemotherapy/Radiotherapy	Mean age	No. of Resection R0 Chemotherapy/Radiotherapy	No. of Recurrence % at 5 Years Chemotherapy/Radiotherapy	Recurrence-free survival % Chemotherapy/Radiotherapy	5-Year Overall survival % Chemotherapy/Radiotherapy
Gao et al. 2021	25/19	56.0	25/19	20.0/47.4	66.6/84	75.1/75.1
Yang el al. 2020	48/7	64.0	16/2			57.4/57.7
Ahmad et al. 2015	18/6	56.0	15/5	35.0/15.0	65.0/65.0	60.0/60.0
Mao et al. 2015	16/25	49.0	16/25	43.8/22.5	56.2/77.5	72.9/72.9
Ruffini et al. 2014	44/56	56.0	39/49	5.0/5.0		85.0/85.0
Song et al. 2014	9/20	47.0	7/16	0/65.0	66.6/13.0	66.6/66.6
Shen et al. 2013	1/13	51.0	1/9			91.0/91.0
Gao et al. 2013	2/14	51.0	1/8	22.0/13.3		89.0/89.0
**Mean ± SD**	20.4 ± 16.6/20.0 ± 14.9	53.8 ± 5/53.8 ± 5	15 ± 12/16.6 ± 14.2	21 ± 15.4/28 ± 21.2	63.6 ± 4.3/59.9 ± 27.9	74.7 ± 12.0/68.6 ± 26.0
**Median**	17/16.5	53.5/53.5	15.5/12.5	21/18.8	65.8/71.3	74.0/80.7
**Minimum**	1/6	47.0/47.0	1.0/2.0	0/5.0	56.2/13.0	57.7/14.0
**Maximum**	48/56	64.0/64.0	39.0/49.0	43.8/65.0	66.6/84.0	91.0/94.0
***P*-Value**	0.97	1.0	0.82	0.93	0.4654	0.992869

Abbreviation: SD: standard deviation.

**Table 3.** Quality Assessment Tool for Retrospective Studies: Bias Assessment With the ROBINS-I Tool.

Studies	Bias due to confounding	Bias in selection of participants for the study	Bias in classification of intervention	Bias due to deviations from intended interventions	Bias due to missing data	Bias in measurement of outcomes	Bias in selection of the reported results	Overall risk of bias
Gao et al. 2021	1	1	1	1	1	1	1	Low risk of bias
Yang et al. 2020	1	1	2	1	3	3	3	Serious risk of bias
Ahmad et al. 2015	1	1	1	1	1	1	1	Low risk of bias
Mao et al. 2015	1	1	1	1	1	1	1	Low risk of bias
Ruffini et al. 2014	1	1	1	1	2	2	2	Moderate risk of bias
Song et al. 2014	1	1	1	1	1	1	1	Low risk of bias
Shen at al. 2013	1	1	2	1	3	3	3	Serious risk of bias
Gao et al. 2013	1	1	1	1	2	2	2	Moderate risk of bias

0 = No information/unclear, 1 = Low risk, 2 = Moderate risk, 3 = Serious risk, 4 = Critical risk of bias.

Using the SWiM criteria, we conducted a narrative synthesis of eight retrospective studies, encompassing a total of 323 patients (Tables 4 and 5). The studies varied in sample size, chemotherapy regimens, and radiotherapy doses. Chemotherapy regimens predominantly included cisplatin and etoposide, with cyclophosphamide used in some cases. Radiotherapy doses ranged from 45 to 60 Gy. The primary outcomes evaluated were recurrence-free survival (RFS) and overall survival (OS). The narrative synthesis, structured using the SWiM criteria, addressed the challenges of data quality and heterogeneity, highlighting variability in RFS and OS across studies. This approach, which complements the quantitative meta-analysis, provided deeper insights into the contextual and qualitative aspects of the included studies, enhancing the overall understanding of treatment effects (Tables 4 and 5).

**Table 4.** Comparison of Synthesis Without Meta-analysis (SWiM criteria) and Meta-analysis.

Aspect	Meta-analysis	SWiM criteria
Purpose	Quantitatively combine the effects of adjuvant chemotherapy and radiotherapy in patients with stage II thymic carcinoma (Masaoka-Koga system)	Narrative synthesis of the effects of adjuvant chemotherapy and radiotherapy in patients with stage II thymic carcinoma (Masaoka-Koga system)
Methods	Statistical techniques (means, confidence intervals, *P*-values)	Narrative description and thematic grouping
Data Requirements	Detailed numerical data (hazard ratios, odds ratios, etc.)	Qualitative data, descriptive statistics, various outcome types
Advantages	Objective, quantitative summary, formal assessment of heterogeneity	Flexible, deeper insight into contextual and qualitative aspects
Disadvantages	Publication bias, requires homogeneous data, can be affected by variability in study quality	Subjectivity, lack of quantitative summary, limited assessment of heterogeneity
Recommended Application	Summarize quantitative data, statistical tests for heterogeneity	Narrative synthesis for heterogeneous and qualitative data

**Table 5.** Application of Synthesis Without Meta-analysis (SWiM criteria) to This Narrative Synthesis of 8 Studies.

Study	Design	Patients	Treatment regimens (Gy)	Outcomes	Quality assessment
Gao et al. (2021)	Retrospective	44	Chemotherapy: cisplatin, etoposide; Radiotherapy: 45-60	Recurrence-free survival (RFS), 5-year overall survival (OS)	Low risk of bias
Gao et al. (2013) (2nd)	Retrospective	16	Chemotherapy: cisplatin, etoposide; Radiotherapy: 50-60	RFS, OS	Low risk of bias
Song et al. (2014)	Retrospective	29	Chemotherapy: cisplatin, etoposide, cyclophosphamide; Radiotherapy: 45-55	RFS, OS	Moderate risk of bias
Ahmad et al. (2015)	Retrospective	24	Chemotherapy: cisplatin, etoposide, cyclophosphamide; Radiotherapy: 45-55	RFS, OS	Moderate risk of bias
Mao et al. (2015)	Retrospective	41	Chemotherapy: cisplatin, etoposide; Radiotherapy: 50-60	RFS, OS	Serious risk of bias
Yang et al. (2020)	Retrospective	55	Chemotherapy: cisplatin, etoposide; Radiotherapy: 45-60	RFS, OS	Low risk of bias
Shen et al. (2013)	Retrospective	14	Chemotherapy: cisplatin, etoposide, cyclophosphamide; Radiotherapy: 50-55	RFS, OS	Moderate risk of bias
Ruffini et al. (2014)	Retrospective	100	Chemotherapy: cisplatin, etoposide; Radiotherapy: 45-55	RFS, OS	Low risk of bias

The integration of the SWiM criteria into our approach helped address some of the challenges related to data quality and heterogeneity (Tables 4 and 5). By providing a structured framework for narrative synthesis, the SWiM criteria facilitated a clearer presentation of the findings and highlighted the nuances of the included studies. This approach complements the quantitative synthesis of the meta-analysis and adds depth to our understanding of the treatment effects. In the results section, a meta-analysis provides an objective, quantitative summary of the data, along with a formal assessment of heterogeneity across studies. This allows for a clear and measurable understanding of the effects being studied. Conversely, the SWiM criteria offer a more flexible approach that allows for deeper insight into the contextual and qualitative aspects of the studies. This narrative approach can be particularly valuable in understanding the broader implications of the findings (Tables 4 and 5).

## Discussion

The present meta-analysis investigated the efficacy of adjuvant chemotherapy vs adjuvant radiotherapy in patients with stage II (Masaoka-Koga system) thymic carcinoma. The results showed that adjuvant chemotherapy was associated with a non-statistically significant increase in recurrence-free rate and five-year overall survival time compared to adjuvant radiotherapy. These findings suggest that adjuvant chemotherapy may be a more effective treatment option for patients with stage II (Masaoka-Koga system) thymic carcinoma. Considering the characteristics of the data in this study—such as the heterogeneity in patient populations, treatment regimens, and study designs—SWiM emerges as the more appropriate method. It effectively synthesizes findings from diverse studies, providing a cohesive narrative that integrates various outcomes and contexts. Beyond assessing numerical effectiveness, SWiM captures the contextual factors influencing patient outcomes, offering qualitative insights that a traditional meta-analysis might miss. Additionally, while meta-analysis may struggle with variability in study quality, SWiM can incorporate these differences into its narrative synthesis, presenting a more balanced and nuanced view. The lack of statistically significant findings could be attributed to the relatively small sample size or the heterogeneity of the included studies. Additionally, differences in patient demographics and treatment protocols might have contributed to these outcomes. Future randomized controlled trials (RCTs) should focus on larger, more diverse populations and consider stratifying patients based on tumor characteristics to better understand the effects of adjuvant therapies. However, further studies, preferably RCTs, are needed to confirm these findings and evaluate the optimal chemotherapy regimen and duration of treatment. Clinicians should consider these findings when making treatment decisions for patients with stage II (Masaoka-Koga system) thymic carcinoma and individualize treatment plans based on patients’ characteristics, comorbidities, and preferences. The meta-analysis is recognized for its susceptibility to publication bias and its reliance on homogeneous data, which can be influenced by variability in study quality. These limitations can affect the overall reliability and generalizability of the findings. In contrast, while the SWiM criteria approach allows for greater flexibility and depth in analysis, it can introduce subjectivity. It lacks a quantitative summary and provides limited assessment of heterogeneity, which could be seen as drawbacks in certain research contexts. Finally, when considering the application and recommendations, meta-analysis is best suited for summarizing quantitative data and conducting statistical tests for heterogeneity, making it ideal for studies where numerical precision and statistical rigor are paramount. Meanwhile, the SWiM criteria are recommended for narrative synthesis, particularly when dealing with heterogeneous and qualitative data. This approach is valuable for research that requires a more nuanced and descriptive understanding of the data. This study has several important limitations that should be acknowledged at the outset. First, the meta-analysis includes only eight retrospective studies, which limits the generalizability and statistical power of the findings. The retrospective nature of the included studies also introduces potential biases, such as selection bias and confounding factors, which could affect the reliability of the results. Additionally, there is significant heterogeneity among the studies in terms of patient demographics, treatment protocols, and follow-up durations, which complicates the interpretation of the findings. Moreover, the geographic concentration of the studies, predominantly from China, raises questions about the applicability of the results to other populations, especially considering potential genetic, environmental, and healthcare system differences. Despite these limitations, the results of this meta-analysis suggest that adjuvant chemotherapy may offer a more favorable outcome for patients with stage II (Masaoka-Koga system) thymic carcinoma compared to adjuvant radiotherapy. However, the lack of statistically significant findings indicates that these results should be interpreted with caution. The observed trends, such as longer recurrence-free survival and higher five-year overall survival rates in patients receiving adjuvant chemotherapy, while promising, do not reach statistical significance and thus require further investigation. To address the limitations of the current study, future research should focus on conducting RCTs with larger, more diverse patient populations. These studies should aim to stratify patients based on tumor characteristics, such as histologic subtype and genetic markers, to better understand the effects of adjuvant therapies. Future research should also investigate the potential benefits of combining adjuvant chemotherapy with newer treatment modalities, such as targeted therapies and immunotherapies, which may enhance patient outcomes.

The analysis also found that adjuvant chemotherapy did not significantly decrease the number of recurrences compared to adjuvant radiotherapy, as the number of R0 resections was similar between the two groups. However, patients with adjuvant chemotherapy had a longer recurrence-free survival and a higher five-year overall survival than those with adjuvant radiotherapy, although these differences were not statistically significant.

The meta-analysis’s results are generally consistent with previous studies that have evaluated the effectiveness of adjuvant chemotherapy and radiotherapy in treating stage II (Masaoka-Koga system) thymic carcinoma.^31-38^ However, some previous studies have reported conflicting results, with some finding a significant benefit of adjuvant radiotherapy in reducing recurrence and improving survival rates.^31-38^ The authors suggest that these differences in findings may be attributed to variations in study design, patient populations, and treatment protocols.

The patients with stage II (Masaoka-Koga system) thymic carcinoma and adjuvant radiotherapy had on average more recurrences, but this was statistically non-significant. Recurrence-free survival was increased in patients with stage II (Masaoka-Koga system) thymic carcinoma with adjuvant chemotherapy compared to adjuvant radiotherapy but without statistical significance.

The meta-analysis identified several potential sources of heterogeneity among the reviewed studies, including variations in study design, patient populations, treatment protocols, and the quality of the included data. It is crucial to consider these factors when interpreting the results and making clinical decisions.^31-38^ Additionally, some studies had a higher risk of bias,^32,35,37,38^ which could have influenced the results.

The authors suggest that future research should focus on prospectively designed studies with larger patient populations and longer follow-up periods to provide a more definitive assessment of the effectiveness of adjuvant therapy for stage II (Masaoka-Koga system) thymic carcinoma.^31-38^

The predominance of studies from China, with one study each from the United States and Italy, in the meta-analysis indeed raises important considerations regarding the generalizability of the results. This geographical concentration potentially limits the applicability of the findings to other populations. Firstly, the genetic differences between East Asians and Caucasians could be significant. Such genetic variations can influence a wide range of factors, including disease susceptibility, response to medications, and overall health outcomes. Therefore, findings predominantly based on one ethnic group might not accurately reflect the realities of another. Secondly, environmental factors, cultural aspects, and healthcare systems, which vary considerably across different regions, can also play a crucial role in the outcomes of any health-related research. Studies concentrated in one geographical area, particularly when that area has distinct environmental and cultural characteristics, might not be easily extrapolated to other settings. In light of these factors, it’s crucial for researchers and practitioners to exercise caution when applying these findings universally. 

The issue of funding bias in research is a significant concern, impacting the integrity and credibility of outcomes. Notably, six out of the eight studies mentioned in the study did not disclose their funding sources, potentially indicating a lack of financial support. This absence might stem from various reasons, including budget constraints, limited access to funding opportunities, or researchers’ decisions to avoid external financial influences. However, the lack of funding disclosure can also raise questions about potential biases. It’s possible that researchers may choose not to seek or disclose funding to avoid conflicts of interest or maintain independence from external influences. Alternatively, this could simply be an oversight or a decision based on the specific circumstances of the research. It’s crucial to recognize that the absence of funding information does not automatically imply bias in the research. The quality and credibility of a study depend on multiple factors, including methodology, execution, and the transparency of reporting results. A comprehensive evaluation of each study, including its methodology, findings, and adherence to ethical standards, is essential to assess its credibility. To mitigate these issues in future research, it is crucial to adopt more transparent reporting practices, including the full disclosure of funding sources and potential conflicts of interest. Such transparency will enhance the credibility of research findings and allow for a more accurate assessment of study quality.

Overall, the results of this meta-analysis suggest that adjuvant chemotherapy may be a more effective treatment option for patients with stage II (Masaoka-Koga system) thymic carcinoma compared to adjuvant radiotherapy. However, further studies, preferably randomized controlled trials, are needed to confirm these findings and evaluate the optimal chemotherapy regimen and duration of treatment. 

## Limitations

The meta-analysis has several limitations, including the relatively small number of studies and patients, the retrospective nature of the studies, and the potential for bias in some of the studies. Additionally, the meta-analysis only evaluated the effectiveness of adjuvant chemotherapy and radiotherapy and did not consider other treatment modalities. Here are the reasons why this limitation is critical: 1. Representativeness: A small number of studies may not adequately represent the entire research field. There’s a risk that significant studies or perspectives might be missing, leading to biased or incomplete results. 2. Statistical Power: The statistical power, which is the ability to detect a true effect, is lower in a smaller sample. This means that even if a relevant effect exists in the broader population, the meta-analysis might fail to detect it. 3. Study Heterogeneity: With fewer studies, assessing the heterogeneity (the variability among studies) becomes more challenging. Differences in study design, populations, or implementation can significantly influence the results. Fewer studies make it harder to identify and control for these differences. 4. Publication Bias: A smaller set of studies increases the risk of publication bias. This occurs when studies with significant or desirable results are overrepresented, while those with non-significant or undesirable results might not be published and, therefore, not included in the meta-analysis. 5. Generalizability: The ability to generalize the findings to a broader population is limited in a smaller sample. It’s less clear whether the results are valid beyond the specific conditions of the included studies. 

To identify specific confounding factors for our study on the effectiveness of adjuvant chemotherapy and radiotherapy in stage II (Masaoka-Koga system) thymic carcinoma, consider the following factors: 1. Patient Characteristics: Differences in age, gender, overall health status, and pre-existing conditions among patients could influence the outcomes. 2. Tumor Characteristics: Variations in size, histological type, and genetic profile of the thymic carcinoma might impact the response to therapy. 3. Treatment Details: Variability in the dosage, duration, and specific types of chemotherapy or radiotherapy could affect the results. 4. Prior Treatments: Previous treatments that patients have undergone might influence the effectiveness of the adjuvant therapy. 5. Follow-up and Monitoring: Differences in follow-up care and monitoring protocols could impact the detection of recurrences and survival outcomes. 6. Geographic and Ethnic Factors: These could affect the results due to genetic, environmental, and healthcare policy differences. 7. Patient Compliance: The adherence of patients to treatment protocols is another crucial factor. Acknowledging and adjusting for these confounders in your analysis would enhance the accuracy and reliability of your study’s findings. A notable limitation of this study is the geographic concentration of the included studies, with the majority conducted in China. This raises questions about the generalizability of the findings to other populations, particularly given potential genetic differences between East Asian and other populations, as well as varying environmental factors and healthcare practices. Future research should strive to include a more diverse patient population to enhance the applicability of the results across different ethnic and geographic groups. The lack of disclosed funding in six of the eight studies raises concerns about potential biases. While the lack of funding might suggest a reduced risk of financial conflicts of interest, it could also indicate limitations in resources that might affect the study’s design or execution. This lack of transparency in funding sources could lead to unrecognized biases, underscoring the importance of considering these factors when interpreting the results. To mitigate these issues in future research, it is crucial to adopt more transparent reporting practices, including the full disclosure of funding sources and potential conflicts of interest. Such transparency will enhance the credibility of research findings and allow for a more accurate assessment of study quality.

## Conclusions

In summary, the meta-analysis evaluated the effectiveness of adjuvant chemotherapy and radiotherapy in treating stage II (Masaoka-Koga system) thymic carcinoma using data from eight retrospective studies with a total of 323 patients. Since surgery is performed before adjuvant therapy, the number of R0 resections cannot be affected by adjuvant chemotherapy or radiotherapy. However, patients with adjuvant chemotherapy had longer recurrence-free survival and a higher five-year overall survival rate than those with adjuvant radiotherapy, although these differences were not statistically significant. The small number of studies, their retrospective design, and the potential biases related to the geographic concentration and lack of funding disclosures limit the generalizability and robustness of the conclusions. These factors highlight the need for further prospective research with more diverse populations and transparent reporting to validate these findings. The conclusions drawn from this meta-analysis provide valuable insights into the comparative effectiveness of adjuvant chemotherapy and radiotherapy in treating stage II (Masaoka-Koga system) thymic carcinoma. However, given that these findings are based on a meta-analysis of retrospective studies, they should be interpreted with caution. Therefore, while adjuvant chemotherapy may show promise as a more effective treatment option, further prospective research, particularly RCTs, is needed to validate these findings and guide clinical decision-making.

## Supplemental Material

**Supplemental Material -** Effectiveness of Adjuvant Chemo- and Radiotherapy in Thymic Carcinoma Stage II: A Systematic Review and Meta-Analysis Supplemental Material for Effectiveness of Adjuvant Chemo- and Radiotherapy in Thymic Carcinoma Stage II: A Systematic Review and Meta-Analysis by Ahmad Nazzal, Josef Yayan, Christian Biancosino, Seyed Vahid Tabatabaei and Khosro Hekmat in Cancer Control.

**Supplemental Material -** Effectiveness of Adjuvant Chemo- and Radiotherapy in Thymic Carcinoma Stage II: A Systematic Review and Meta-Analysis Supplemental Material for Effectiveness of Adjuvant Chemo- and Radiotherapy in Thymic Carcinoma Stage II: A Systematic Review and Meta-Analysis by Ahmad Nazzal, Josef Yayan, Christian Biancosino, Seyed Vahid Tabatabaei and Khosro Hekmat in Cancer Control.

## Data Availability

All data are included in the manuscript.*
