# Supplementary material for: Effectiveness of Adjuvant Chemo- and Radiotherapy in Thymic Carcinoma Stage II: A Systematic Review and Meta-Analysis
Source: Cancer Control. 2024 Oct 17;31:10732748241292781. doi: 10.1177/10732748241292781 (PMC11503756; doi:10.1177/10732748241292781)
Supplement: Supplemental Material - Effectiveness of Adjuvant Chemo- and Radiotherapy in Thymic Carcinoma Stage II: A Systematic Review and Meta-Analysis [file sj-pdf-1-ccx-10.1177_10732748241292781.pdf]

Data Collection and Presentation Checklist:

The review of the data collection and presentation checklist has been completed.

**Ethical Statements (if applicable):**		
**Humans**	Complete and upload the Ethics Declaration Statement you received with your decision letter. **checked**	☐
**Animals (NONE)**	Provide species, sex, strain, age, source and husbandry conditions. **checked**	☐
	Note if the study was blinded or not. **checked**	☐
	Provide a statement confirming the research was approved by the Institutional Animal Care and Use Committee. **checked**	☐
	Complete and upload the ARRIVE Compliance Questionnaire. Visit ARRIVE for more information. **checked**	☐
**Reagents and Biological Materials: (NONE)**		
	Include manufacture name, catalog number (and lot number for antibodies) for all reagents used (including fluorochromes and stains). **checked**	☐
	Cell lines: provide source, derivation and authentication method. **checked**	☐
**Images**		
**General**	Do not introduce or remove any features in your images. Leave any blemishes. **checked**	☐
	If any adjustments to contrast, balance or brightness are made, they must be applied uniformly across the entire image. Any nonlinear adjustments must be disclosed in the legend. **checked**	☐
	Check that images are not pixelated when reasonably magnified. Images must be at 300 dpi. TIFF images are encouraged. Avoid jpegs or using PowerPoint as this will compress your images. **checked**	☐
	Scale bars must be included. **checked**	☐
	If splicing images, the borders must be marked and noted in the legend.	☐

**Microscopy (include the following) (NONE)**	Camera make and model. **checked**	☐
	Microscope make and model. **checked**	☐
	Objective magnification, type and numerical aperture. Magnification must be mentioned in figure legend. **checked**	☐
	Fluorochromes and stains. They should also be mentioned in the legend. **checked**	☐
	Acquisition software. **checked**	☐
	Show all individual channels in grey scale and merged image in color (all at the same intensity). **checked**	☐
**Western Blots (NONE)**	Westerns should not be modified for contrast, the entire tonal range should be present. **checked**	☐
	Include at least two molecular weight markers, one above and one below your band of interest. **checked**	☐
	If a blot is spliced together, you must mark the border and explain this in the legend. Splicing across different blots is not allowed. **checked**	☐
	It is best practice to normalize protein levels against total protein, not house-keeping proteins. **checked**	☐
	Post-translationally modified proteins (PTMs) must use total protein for normalization. **checked**	☐
	Provide raw blots as supplementary data. These may be combined as a single Word doc. Blots must be accurately labeled to match figures in the main doc. Include the molecular weight ladder. **checked**	☐
**RNAi, Gene Expression, Microarrays (NONE)**	At least two different siRNAs targeting different gene areas must be used. **checked**	☐
	At least two different control siRNAs must be used. **checked**	☐
	Gene expression studies cannot be presented alone without providing evidence that the changes in levels have downstream functional consequences. **checked**	☐
	Microarray data must include:<br>○ The raw data for each hybridization.<br>○ Experimental Factors and values.<br>○ Experimental design.<br>○ Data processing protocols (e.g., normalization method).	☐

**Cell culture (NONE)**	At least three appropriate cell lines should be used to confirm findings. If there are fewer, add a statement explaining why only 1 or 2 were used. **checked**	☐
